# An electrically conductive dinuclear double helicate Al(iii) complex for the fabrication of a Schottky diode

**DOI:** 10.1039/d6ra03765h

**Published:** 2026-07-22

**Authors:** Md Hasan Raja, Sayantan Sil, Samim Ahmed, Ramjan Sk, Md Rafikul Alam Mondal, K. Mohamed Yusuf Baig, Manash Pratim Sarmah, Nargis Khatun, Partha Pratim Ray, Goutam Kumar Kole, Manabendra Sarma, A. K. M. Maidul Islam, Ennio Zangrando, Md. Akhtarul Alam

**Affiliations:** a Department of Chemistry, Aliah University Action Area IIA/27, New Town Kolkata-700160 India alam_iitg@yahoo.com alam@aliah.ac.in; b Department of Basic Science and Humanities, Institute of Engineering and Management, University of Engineering and Management, Kolkata, University Area Action Area III, B/5, New Town Kolkata 700160 India; c Department of Physics, Jadavpur University Kolkata 700032 India partha@phys.jdvu.ac.in; d Department of Chemistry, SRM Institute of Science and Technology Kattankulathur Tamil Nadu 603203 India goutamks@srmist.edu.in gkkole@gmail.com; e Department of Chemistry, Indian Institute of Technology Guwahati Assam-781039 India msarma@iitg.ac.in; f Department of Physics, Aliah University Action Area IIA/27, New Town Kolkata-700160 India maidul79@gmail.com; g Department of Chemical and Pharmaceutical Sciences, University of Trieste Via L. Giorgieri 1 34127 Trieste Italy ezangrando@units.it

## Abstract

The crystallographic elucidation of a dinuclear aluminium(III) helicate (1) consisting of the biphenyl-derived two-arm-containing amido Schiff base ligand bis(2-hydroxybenzylidene)-[1,1′-biphenyl]-2,2′-dicarbohydrazide (ligand H_4_L) is reported. The X-ray single crystal diffraction studies of complex 1 reveal that it crystallizes in the triclinic space group *P*-1. Each Al^3+^ ion is coordinated to four oxygen and two nitrogen atoms from the two terdentate ligands to form an octahedral geometry. Furthermore, several types of intra-/inter-molecular interactions, including H-bonding, C–H⋯O weak interactions, and π⋯π interactions, stabilize its crystal lattice, leading to a supramolecular polymeric structure. The electrical performances of H_4_L and 1 are investigated in a metal–semiconductor (MS) junction. A significant enhancement in electrical conductivity and improved Schottky barrier diode (SBD) parameters are observed for 1 compared to its precursors. The *I*–*V* characteristics demonstrated that, upon complexation with the Al(iii) ion, conductivity increased by twice the magnitude (H_4_L: 4.02 × 10^−4^ Sm^−1^; complex 1: 8.70 × 10^−4^ Sm^−1^) with an improved diode rectification ratio, which can be corroborated to the supramolecular polymeric structure of 1. Measurements of the optical bandgap as well as the HOMO–LUMO gap computed by density functional theory (DFT) calculations confirm the semiconducting properties of 1. Moreover, the theoretical DOS/PDOS plots provide evidence for the Schottky diode function of the complex.

## Abstract 2

The electrical conductivity of the Al(iii) complex is recorded under dark conditions, and the complex exhibited the characteristics of a Schottky barrier diode (SBD).

## Graphical abstract

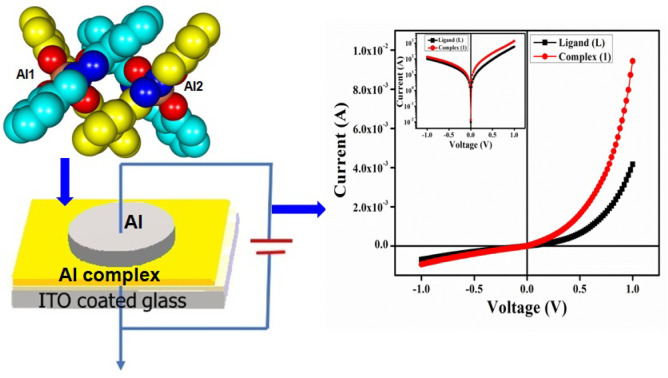

## 1. Introduction

Over a few decades, scientists have significantly contributed towards the synthesis of a variety of Schiff bases, commonly available with N and O donor atoms, which are useful for the syntheses of covalent organic frameworks (COFs),^1–3^ metal organic frameworks^4–7^ (MOFs), coordination polymers^8,9^ (CPs) and coordination complexes (CCs).^10–13^ These Schiff base-derived compounds, prepared in different experimental conditions using a variety of molecular precursors (amines and aldehydes), exhibit exceptional thermal stability and an extensive range of attractive physico-chemical properties for versatile potential applications in different fields of materials science. Examples of their applications include gas storage,^14,15^ separation including gas,^16^ hydrocarbons and other liquids,^17,18^ CO_2_ capture,^19^ ion exchange,^20,21^ magnetic material design,^22–24^ catalysis,^25–27^ drug delivery,^28–30^ manufacturing of semiconductive and photoconductive devices,^31,32^ and sensing of target molecules.^33–35^

Furthermore, in recent years, CCs with Schiff bases have been shown to conduct electricity,^36–38^ with diverse applications in electronics^39,40^ and optoelectronics,^41^ specifically as Schottky barrier diodes (SBDs).^42^ SBDs have found widespread use in various fields, such as photovoltaics, transistors, supercapacitors, batteries, and sensors, making them a current research topic.^43–48^

Numerous transition metal complexes with conjugated Schiff base ligands have been reported to exhibit fascinating electrical conductivity properties.^49^ For instance, cadmium(ii),^50–52^ zinc(ii),^53,54^ and copper(ii)^55–57^ complexes with Schiff bases are remarkable examples of conductivity-based photo-switching devices. Cd(ii)-based coordination polymers reported by Majumdar *et al.*^58^ and by Saha *et al.*^59^ exhibit photosensitive Schottky barrier diode behavior. Dong *et al.* reported that Ag(i) complexes containing a double Schiff-base ligand can exhibit luminescent and electrical conductive properties.^60^ In addition, hetero-metal complexes^61,62^ containing Schiff base ligands have been used to fabricate photosensitive Schottky barrier diodes and semiconductor devices.

Despite these examples, Al(iii) complexes with amido Schiff bases and their electrical conducting properties have been less explored.^63,64^ In the case of Al^3+^, a hard acid ion, sometimes its preferred six coordination cannot be satisfied by ordinary Schiff bases with two hard bases, either N/O or N/N.

As for ligands, amido Schiff bases are a class of versatile molecules capable of forming different molecular architectures due to the amide group containing self-complementary H-bond donor and acceptor sites. Moreover, amide oxygen bonded metal complexes are particularly attractive for studying the influence of the amide proton on interactions with metal ions. Interestingly, Li *et al.* reported the replacement of the hydrogen atom in the –NHCO– group with a “magic methyl”, a strategy that effectively manipulated the photoluminescence and photochromism of salicylaldehyde benzoylhydrazones in the solid state.^65^ By continuing our studies on metal–organic assemblies,^66,67^ our designed amido Schiff base ligands with rich oxygen and nitrogen donors can offer suitable binding sites to form chelate compounds with Al^3+^ ions.

Considering these premises, we have synthesised a biphenyl-derived two-arm-containing amido Schiff base ligand, bis(2-hydroxybenzylidene)-[1,1′-biphenyl]-2,2′-dicarbohydrazide (H_4_L), and its Al(iii) complex (1). The single-crystal X-ray analysis revealed the formation of a dinuclear Al_2_L_2_ helicate, where the two deprotonated terdentate ligands coordinate each Al(iii) through the amide oxygen, imino nitrogen and phenolic oxygen donors. The electrical conducting properties of the ligand H_4_L and its Al(iii) complex (1) indicate Schottky barrier diode behaviour for the latter. The optical band gaps of 1 and H_4_L were also determined and compared with the DFT-computed values, confirming the Schottky barrier diode behavior of 1.

## 2. Experimental section

### 2.1. Materials

All reagents and spectroscopic grade solvents were used as received from commercial sources without further purification. Al(iii) nitrate salt was purchased from Sigma-Aldrich Chemical Company. Aqueous medium experiments were performed in deionized water.

### 2.2. Methods

UV-vis absorption spectra were recorded on a Shimadzu UV-1900 spectrophotometer. FT-IR spectra (KBr pellet, 4000–400 cm^−1^) were recorded on a PerkinElmer model 883 spectrophotometer. The conductance of the solution was measured using a D-511 digital conductivity meter by Digital Instrument Corporation, India. Elemental analyses (C, H, N) were performed using a CHNS-O 2400II PerkinElmer elemental analyzer. Powder X-ray diffraction (PXRD) patterns were recorded using a PANalytical X'Pert Pro diffractometer with Cu-Kα radiation (*λ* = 1.5418 Å) at a scan speed of 2° min^−1^. The thermogravimetric analyses (TGA) of complex 1 were performed using a PerkinElmer Pyris-1 Thermogravimetric Analyser with a heating rate of 5 °C per min in a N_2_ atmosphere.

### 2.3. Theoretical methods

All the optimization calculations were performed at the DFT-B3LYP/6-311G(d) level using Gaussian-16 software.^68^ The DOS/PDOS calculations were performed using Quantum ESPRESSO^69^ software to analyze the conductivity of the compounds. The Perdew–Burke–Ernzerhof (PBE) function with generalized gradient approximation (GGA) was used to approximate the exchange–correlation terms.^70^ Projector augmented-wave (PAW) pseudo potential treats the electron-ion core interaction by maintaining the cutoff energy for the plane wave at 200 eV. For the self-consistent field (SCF) calculation, the Brillouin zone was sampled at 3 × 3 × 3 and the DOS calculation at 6 × 6 × 6.

### 2.4. Device fabrication

For the comparative electrical study, metal–semiconductor (MS) junction devices were fabricated by sandwiching the active material (either H_4_L or complex 1) between indium tin oxide (ITO) and Al electrodes. The fabrication involved preparing a 5 mg mL^−1^ dispersion in *N*,*N*-dimethyl formamide (DMF) medium which was then applied to ITO substrates *via* spin-coating using an SCU 2700 spin coater unit. Each device underwent four consecutive coating cycles at 600 rpm for 60 seconds to establish the active semiconducting layer. After drying under vacuum, the film thickness was determined to be 1 µm. Aluminium top electrodes were evaporated at 10^−6^ Torr using a HindHivac Vacuum Coating Unit 12A4D, creating a defined contact area of 7.065 × 10^−6^ m^−2^ using a shadow mask. The resulting devices were then analyzed using a Keithley 2635B source meter with a computer controlled two-probe configuration to record current–voltage (*I*–*V*) characteristics between −1 V and +1 V under dark conditions at room temperature.

### 2.5. X-ray crystallography

An appropriate single crystal of complex 1 was mounted on a Bruker–AXS SMART APEX II diffractometer equipped with graphite monochromated Mo-Kα (*λ* = 0.71073 Å) radiation. The SAINT package was used for data collection and reduction, while absorption correction was applied using the SADABS program.^71^ The structure was solved using SHELXT 2014/4 (ref. 72) and subsequent difference Fourier syntheses. Non-hydrogen atoms were refined with anisotropic displacement parameters (except for the disordered lattice DMF molecule). Hydrogen atoms were placed in idealized positions and their displacement parameters were fixed 1.2 times larger than the thermal factor of the atom to which they were attached. CCDC deposition no. 2515568 contains the supplementary crystallographic data for this paper.

### 2.6. Synthesis of ligand (H_4_L)

Ligand H_4_**L**, bis(2-hydroxybenzylidene)-[1,1′-biphenyl]-2,2′-dicarbohydrazide was synthesized by dropwise addition of a methanolic solution of 2-hydroxy benzaldehyde into a methanolic solution of [1,1′-biphenyl]-2,2′-dicarbohydrazide according to the reported procedure^66^ and was used without further characterization (Fig. S1). The single crystal structure has already been deposited as CCDC 1990271.

### 2.7. Synthesis of complex [Al_2_(L)(H_2_L)]·3H_2_O·DMF (1)

A methanolic solution of Al(NO_3_)_3_·9H_2_O (0.267 mmol, 100 mg) was added to a methanolic solution of H_4_L (0.267 mmol, 128 mg) and stirred for 2 h. The as-formed light yellow precipitate was filtered and washed with methanol, dissolved in DMF and subjected to ether diffusion. After four days, light yellow rod-shaped crystals of 1 were obtained. Yield: 87 mg, 50%. Elemental analysis calcd (%) for C_59_H_51_Al_2_N_9_O_12_: C 62.60, H 4.54, N 11.14; found C 62.32, H 4.65, N 10.89. FT-IR (KBr): 3426, 1617, 1552, 1507, 1387, 1334, 1207, 1149, 890, 756, 698 cm^−1^ (Fig. S7). *Λ*_M_: (DMF) 5 Scm^−2^ mol^−1^. ESI-MS (positive mode, *m*/*z*). Calcd for C_56_H_39_Al_2_N_8_O_8_: 1005.92, found: 1005.22 (Al_2_L_2_ + H^+^); C_32_H_29_Al_2_N_6_O_6_: 647.18, found: 647.46 (Al_2_L·2CH_3_CN·2H_2_O); C_28_H_20_AlN_4_O_4_: 503.13, found: 503.13 (AlL) (Fig. S8).

## 3. Results and discussion

### 3.1. Crystal structure of complex 1

**Fig. 1:**
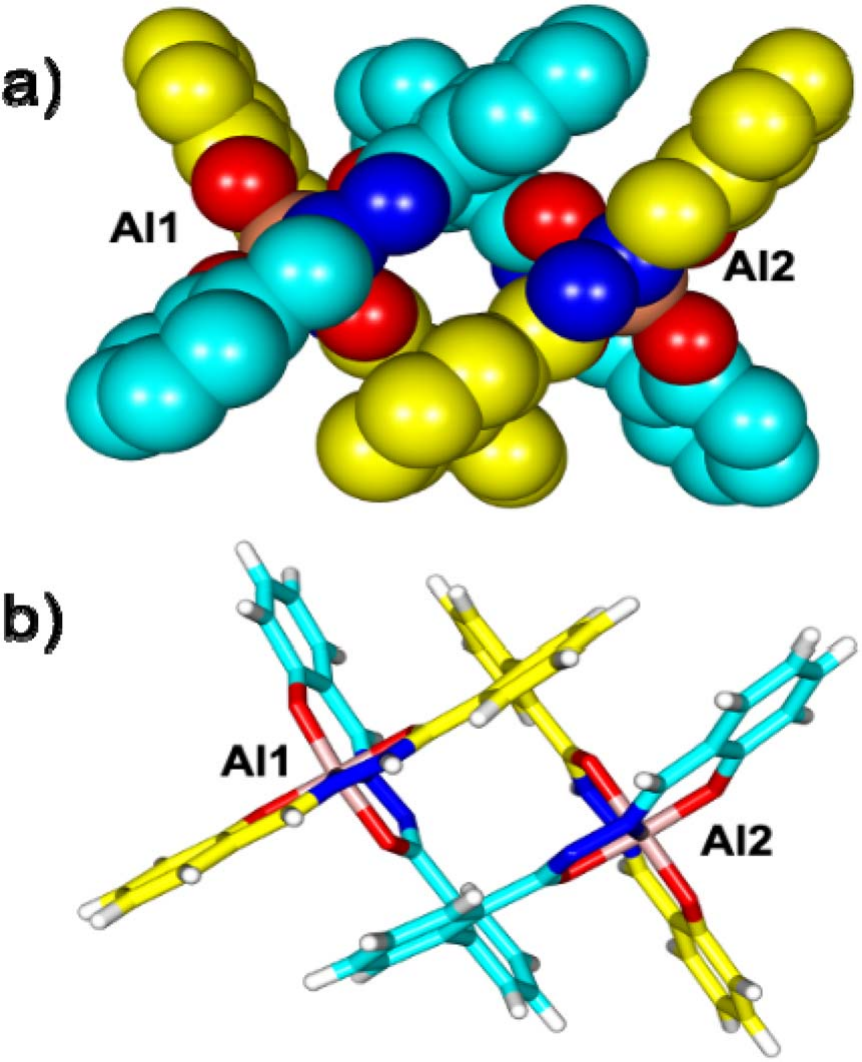
Helicate structure of complex 1: (a) space fill and (b) stick model. Solvent molecules are omitted for clarity.

Single crystal diffraction studies of complex 1 revealed that the complex crystallizes in the triclinic space group *P*1̄ of [Al_2_(L)(H_2_L)]·3H_2_O·DMF. It consists of two deprotonated ligands (L) bearing two-armed terdentate donor sites wrapping two six-coordinated Al^3+^ ions, one DMF and three H_2_O solvate molecules. A model of the complex is shown in Fig. 1, while an ORTEP representation is shown in Fig. S2. The detailed crystallographic data and selected bond lengths and angles are summarized in Tables S1–S3 (in the SI), respectively.

The bis-terdentate ligands L are meridionally coordinated, acting as bridging ligand with the two pocket-like coordination sites at the terminal. The complex is best described as a double stranded helicate, with the two Al(iii) centers occupying the helical axis separated by 6.519 Å (Fig. 1). This distance is *ca.* 0.7 Å longer than that measured in the comparable structure [NaAl_2_(L)_2_]·2H_2_O·4DMF reported from our laboratory.^63^

**Fig. 2:**
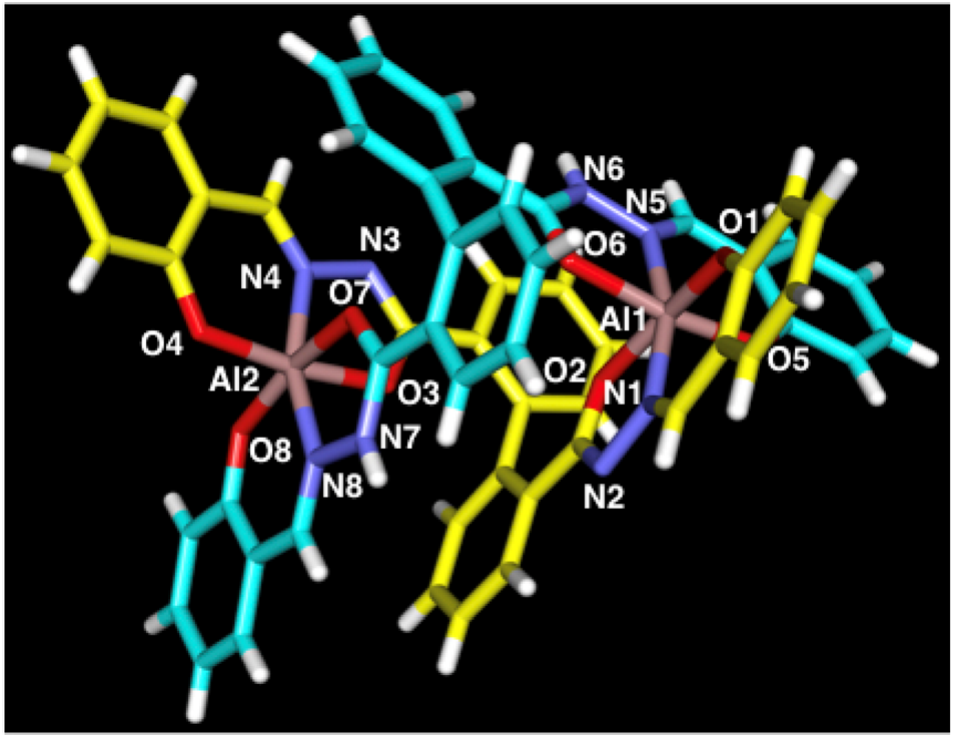
Perspective view of the dinuclear Al(iii) complex 1 with partial atom labels.

In 1, the hexa-coordinated Al1 atom is coordinated by two phenolate oxygens (Al1–O1 = 1.8221(19), Al1–O5 = 1.8162(19) Å), two amide oxygens (Al1–O2 = 1.8742(19), Al1–O6 = 1.9466(18) Å) and two imino nitrogens (Al1–N1 = 1.982(2), Al1–N5 = 2.013(2) Å) from both ligands (Fig. 2). Similarly, the hexacoordinated Al2 atom possesses an O_4_N_2_ donor set from the other two arms of the ligands (Al2–O4 = 1.8312(18), Al2–O8 = 1.8222(18), Al2–O3 = 1.8890(19), Al2–O7 = 1.9664(18), Al2–N4 = 1.967(2), Al2–N8 = 1.999(2) Å (Tables S2 and S3)).

The coordinated amides exhibit interesting features, as one group shows a different behaviour compared with the other.^73^ The following interesting features are worth noting:

(i) The Al1–O6(amide) bond length of 1.9466(18) Å is relatively longer than the Al1–O2(amide) bond of 1.8742(19) Å of the other ligand. Similarly, the Al2–O7(amide) bond length is 1.9664(18)Å, slightly elongated compared to the Al2–O3(amide) of 1.8890(19) Å (Table 1 and Fig. S3). This feature likely favors the coordination and bridging behaviour of the ligands towards Al atoms.

**Table 1:** Al–O, amide C=O and amide C–N bond lengths (Å) in complex 1 and ligand H_4_L^70^

	–C=O⋯Al^3+^	–C=O	Carbonyl–amide –C–NH
Ligand H_4_L	—	1.222(2)	1.339(3)
		1.233(2)	1.334(2)
Al1	1.8742(19)	1.289(3)	1.321(3)
	1.9466(18)	1.247(3)	1.338(3)
Al2	1.8890(19)	1.283(3)	1.325(3)
	1.9664(18)	1.254(3)	1.336(3)

(ii) In complex 1, the amide group containing C36–O6 (carbonyl) bond distance is 1.247(3) Å, almost close to the values measured in the free ligand H_4_L (–C=O in of 1.222(2) and 1.233(2) Å, Table 1).^66^ On the other hand, the carbonyl C8–O2 bond distance of 1.289(3) Å is about 0.062 Å higher than the mean value of the –C=O double bond character in the free ligand H_4_L (Table 1). Correspondingly, the Al1–O2 bond distance is shorter (stronger) than Al1–O6.

A similar trend is observed for the C49–O7 and C21–O3 carbonyl bond distances of 1.254(3) and 1.283(3) Å, respectively. Again, the Al2–O7 and Al2–O3 bond distances follow an inverse order (Table 1).

(iii) Making allowance for the electrostatic discharge (ESD), a short carbonyl amide C–NH bond appears to be associated with a longer C=O bond of the corresponding amide and *vice versa*. At Al1, the C8–N2 bond distance of 1.321(3) Å is related to the amide C8–O2 of 1.289(3) Å, as is the slightly longer C36–N6 of 1.338(3) Å to the shorter amide C36–O6 of 1.247(3) Å. A comparable trend is also observed for Al2 (Table 1).

**Fig. 3:**
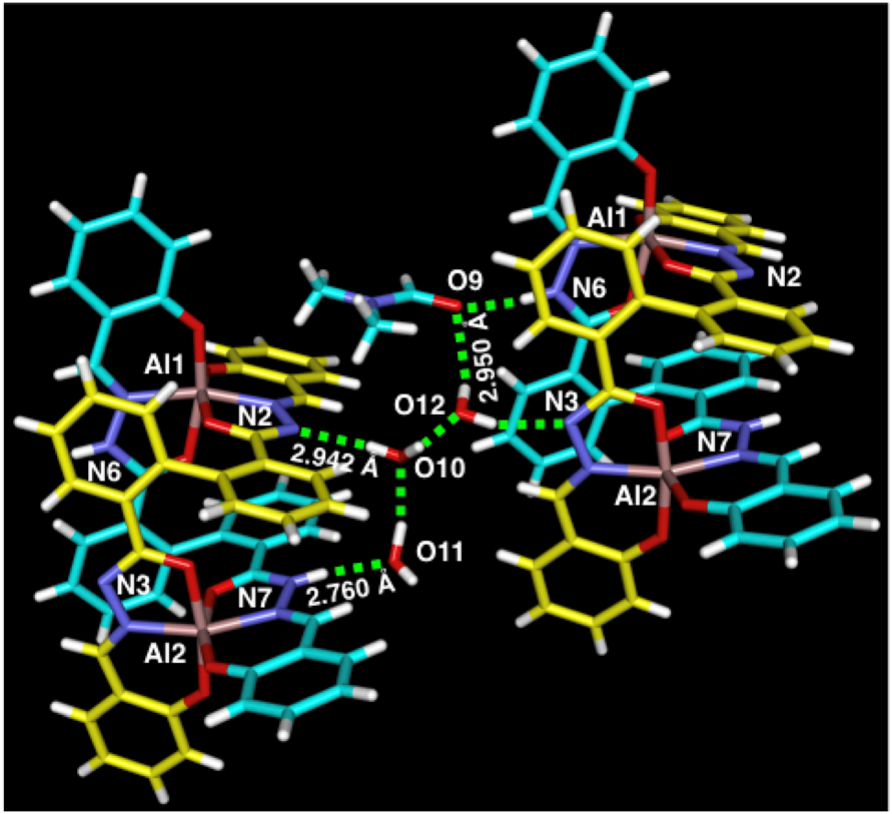
Solvent molecules act as a bridge to form a dimer between two Al^3+^ complexes.

**Fig. 4:**
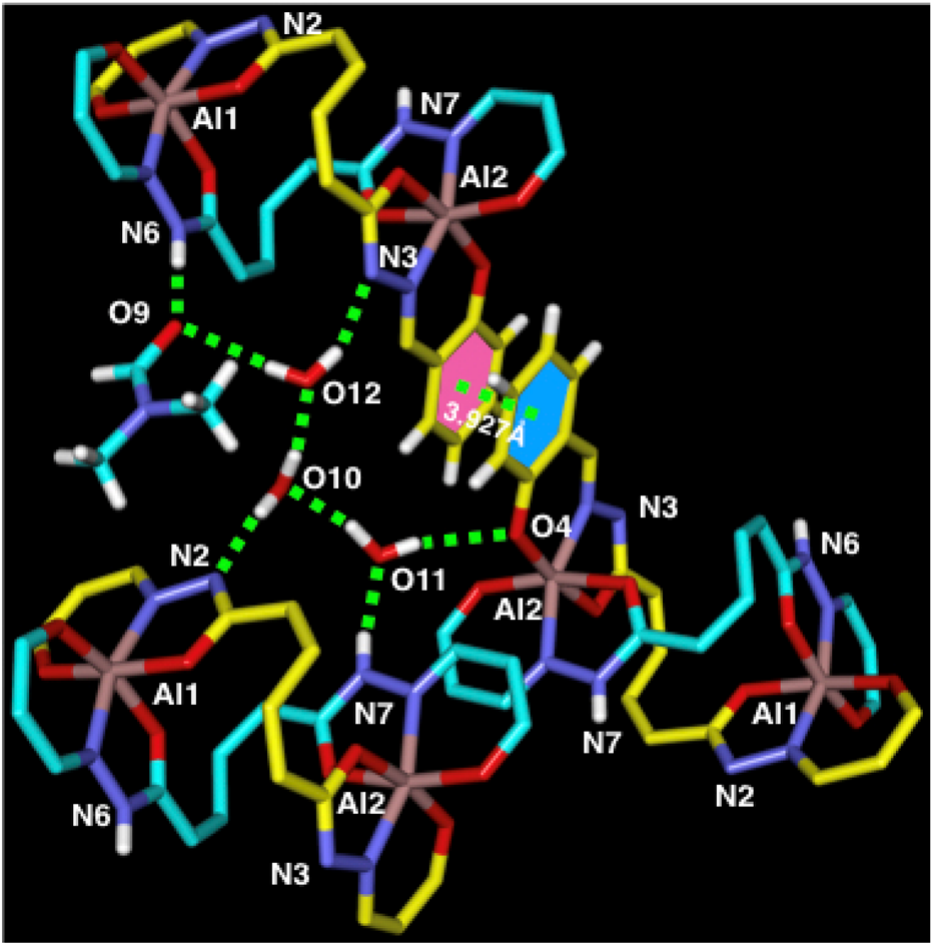
Partial representation of coordination sphere around Al^3+^ in complex 1; H-bonding and π–π interactions of three units of complex 1.

The above observed trend for the Al–O, carbonyl C=O and amide C–N bond lengths clearly indicates that, in complex 1 among the four arms of the two ligands, only two amide groups are deprotonated (–CO–N^−^). Close observation reveals that only one ligand evidences both amide groups being deprotonated (Fig. 3 and 4, yellow colour ligand L) leading to L^4−^, and in order to guarantee the electroneutrality of the Al(iii) complex, the other ligand is di-deprotoned (H_2_L^2−^) giving rise to the correct formulation of [Al_2_(H_2_L)(L)]. Such type of coordination behaviour of the amide groups, which are strong sigma donors and are known to stabilise metal ions in high oxidation states, is scarcely reported in the literature.^74^

The crystal lattice contains one DMF and three water molecules. As a matter of fact, the amide group containing N6–H6 is involved in strong hydrogen bonding with the DMF oxygen atom O9 (2.875 Å) (Fig. 3 and 4). The water molecule O12 acts as a H-bond donor towards the DMF oxygen O9 (O12⋯O9 = 2.950(5) Å) and the deprotonated amide nitrogen N3 atom (O12⋯N3 = 2.818(6) Å) in addition to behaving as a H-bond acceptor from the water molecule O10 (O10⋯O12 distance of 2.651 Å). Similarly, the water molecule O10 is H-bonded to the deprotonated amide atom N2 of the ligand (O10⋯N2 = 2.942(4) Å). The third water molecule O11 interacts *via* H-bond with O10 (O11⋯O10 = 2.732(4) Å), and with the phenolate oxygen O4 of a symmetry related complex (O11⋯O4 = 2.982(3) Å). Finally, amide group N7–H strongly interacts with water O11 (N7⋯O11 = 2.760(4) Å). All H-bond parameters are reported in Table S4.

In addition, the crystal lattice is stabilized by π⋯π interactions between molecular units forming a 1D chain (Fig. S4 and Table S8) and C–H⋯π weak interactions between molecular units that give rise to a 2D supramolecular network (Fig. S5 and Table S7). Furthermore, C–H⋯O weak interactions between molecular units and solvent molecules are also observed (Fig. S6 and Table S6).

The powder X-ray diffraction (PXRD) analysis of complex 1 revealed a pattern matching the simulated one from the single-crystal structural data, suggesting the presence of common structural elements in the powder and in the single crystal of complex 1 (Fig. S9).

Further, to clarify the exact number of solvent molecules per formula unit of complex 1, we carried out thermogravimetric analysis (TGA), which exhibits a curve that corresponds to a weight loss of 4.8% in the 80–150 °C range, corresponding to the loss of three water molecules, and a further 6.5% loss in the 170–270 °C temperature range, which corresponds to the loss of one DMF molecule (Fig. S10). The total weight loss observed (11.3%) matched well with the expected weight loss of 11.2%, corresponding to three water molecules and one DMF molecule, confirming that the composition of complex 1 is [Al_2_(L)(H_2_L)]·3H_2_O·DMF. Further, complex 1 exhibited good thermal stability up to 400 °C.

## 4. Electrical characterisation: *I*–*V* analysis

To evaluate the device performance, electrical characterizations were conducted on thin film metal–semiconductor (MS) junction devices. Schottky diodes were constructed in an Al/active material/ITO sandwich structure, separately employing the pure ligand H_4_L and complex 1 as the active semiconducting material.

**Fig. 5:**
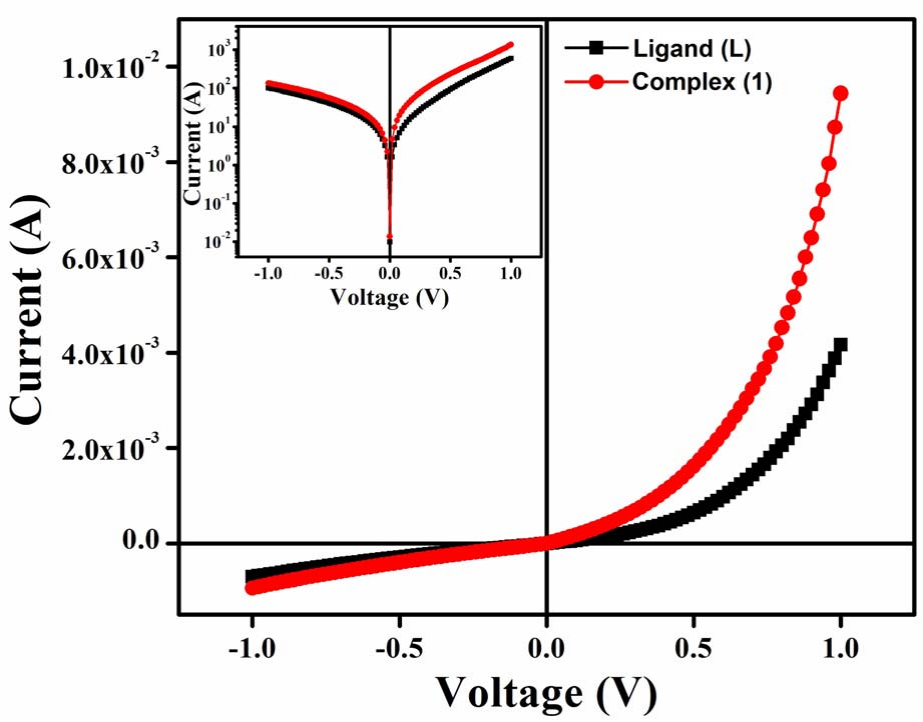
*I*–*V* characteristics of Al/complex 1/ITO and Al/ligand H_4_L/ITO in linear scale and semi-logarithmic scale (inset).

The *I*–*V* measurements were performed in the dark at room temperature over a bias range of ±1.0 V, and the resulting current–voltage (*I*–*V*) profiles for the ligand- and complex-based devices are illustrated in Fig. 5. As evident from the plot, complex 1 shows a higher current magnitude than the free ligand. The dark conductivities were calculated to be 4.02 × 10^−4^ Sm^−1^ for ligand H_4_L and 8.70 × 10^−4^ Sm^−1^ for complex 1. The inset of Fig. 5 displays the corresponding logarithmic *I*–*V* plot.

It is worth noting that complex 1 exhibits a current flow approximately one order of magnitude higher than that of the ligand L. This observation suggests that the coordination of the Al(iii) center substantially enhances the overall conductivity of the material. The formation of the 1D supramolecular architecture *via* C–H⋯π interactions, described above, improves the overall conductivity and more efficiently promotes the electron transfer than ligand H_4_L. The improved conductivity of metal complex 1 is also ascribed to its optimal band gap, as demonstrated by UV-vis analyses and DFT computations.

To gain a deeper understanding of how charge carriers move through the devices, the thermionic emission (TE) model was employed.^75^ Based on this theoretical framework, the diode current can be expressed using the following relation,^76,77^1
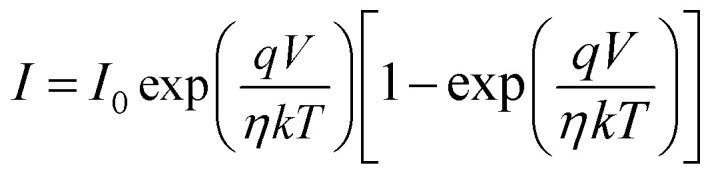
where2
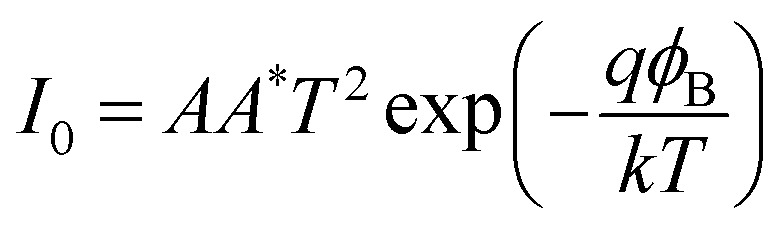
3
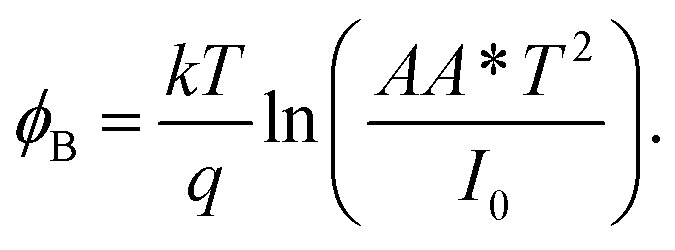
Here, *I*_0_ represents the saturation current, *q* is the elementary electronic charge, *k* represents the Boltzmann constant and *T* is the absolute temperature in Kelvin. The term *V* refers to the applied forward bias voltage, while *η* and *ϕ*_B_ denote the ideality factor and the effective barrier height at zero bias, respectively. The active diode area, *A*, is defined as 7.065 × 10^−6^ m^2^, and the effective Richardson constant, A*, is taken to be 1.20 × 10^6^ Am^−2^ K^−2^. To account for the influence of series resistance on the forward bias *I*–*V* characteristics, Cheung's method is applied, yielding the following expression.^78^4
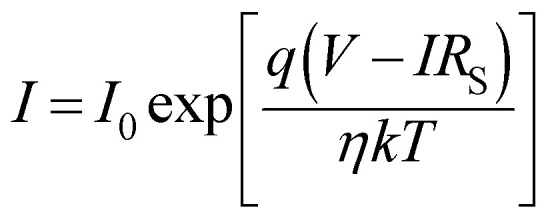


The voltage drop due to the series resistance of the device is denoted by *IR*_S_. By applying this parameter, the series resistance can be calculated through the following functional relationships derived from eqn (4).^79^5
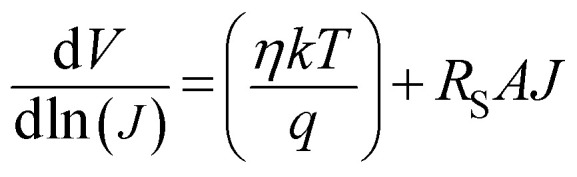

6 *H*(*J*) = *R*_s_*AJ* + *ηϕ*_B_

*H*(*J*) can be expressed as follows.7
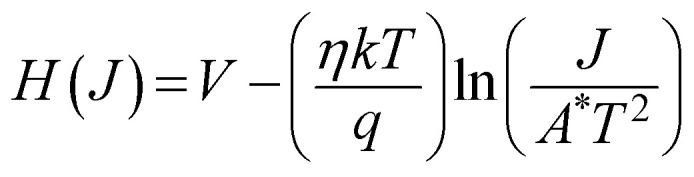


**Fig. 6:**
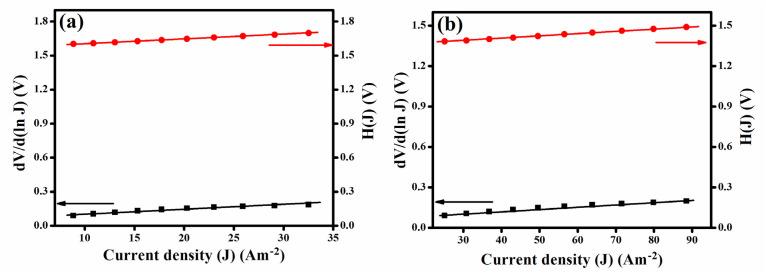
d*V*/d(ln *J*) *vs. J* and *H*(*J*) *vs. J* curves for (a) ligand H_4_L and (b) complex 1 under dark conditions.

The values for the series resistance (*R*_S_) and ideality factor (*η*) under dark conditions for the devices were extracted from the slope and intercept of the d*V*/dln(*J*) *versus J* plot under dark conditions, as shown in Fig. 6. Additionally, the barrier height for each junction was determined using the *y*-axis intercept of *H*(*J*) *versus J* curve (Fig. 6). The *H*(*J*) plot provides a secondary calculation for series resistance *via* its slope. All calculated parameters, including the ideality factor, barrier height and series resistance, are summarized in Table 2.

**Table 2:** Schottky diode parameters of ligand H_4_L and complex 1

Sample	Measured condition	Conductivity (*δ*) (S m^−1^)	Rectification ratio	d(*V*)/d(ln *J*) *vs. J* graph		*H*(*J*) *vs. J* graph	
				Ideality factor (*η*)	Series resistance (*R*_s_) (Ohm)	Barrier height (*ϕ*_b_) (eV)	Series resistance (*R*_s_) (Ohm)
LigandH_4_L	Dark	4.02 × 10^−4^	5.98	2.50	564.75	0.62	588.1
Complex 1	Dark	8.70 × 10^−4^	7.40	2.23	236.37	0.59	234.45

The calculated ideality factor (*η*) values deviate from unity under dark conditions. This deviation from the ideal case is likely a result of the combined effects of series resistance, interface state density and barrier height inhomogeneity. Furthermore, enhanced recombination of charge carriers within the depletion region may also contribute to this deviation.^80,81^

**Fig. 7:**
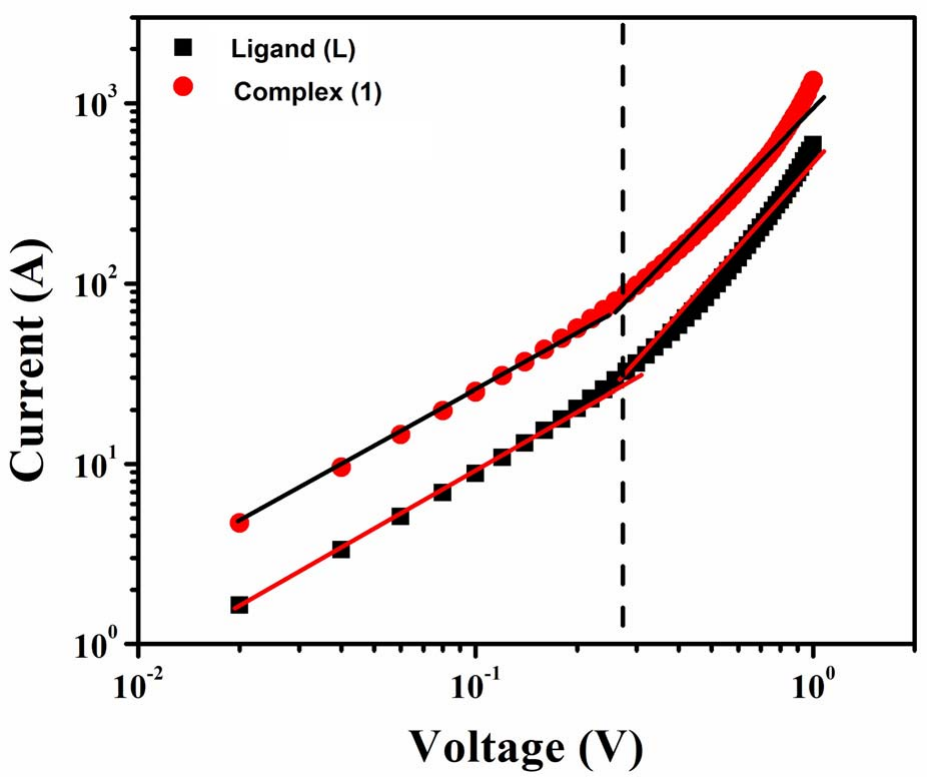
Logarithmic plots of *I*–*V* characteristics under dark conditions for the H_4_L ligand and complex 1.

The transport properties of devices are characterized by a power law (*I* ∞ *V*^*m*^),^82^ where *m* represents the slope of the *I*–*V* plot. As illustrated in Fig. 7, two distinct conduction modes were observed under forward bias. At low voltages (Region I), the behavior is strictly ohmic (*m* ≈ 1). However, at higher potentials (Region II), the current follows a quadratic dependence on voltage (*I* ∞ *V*^2^), identifying the space charge limited current (SCLC) model with discrete traps as the primary conduction mechanism.^83,84^

**Fig. 8:**
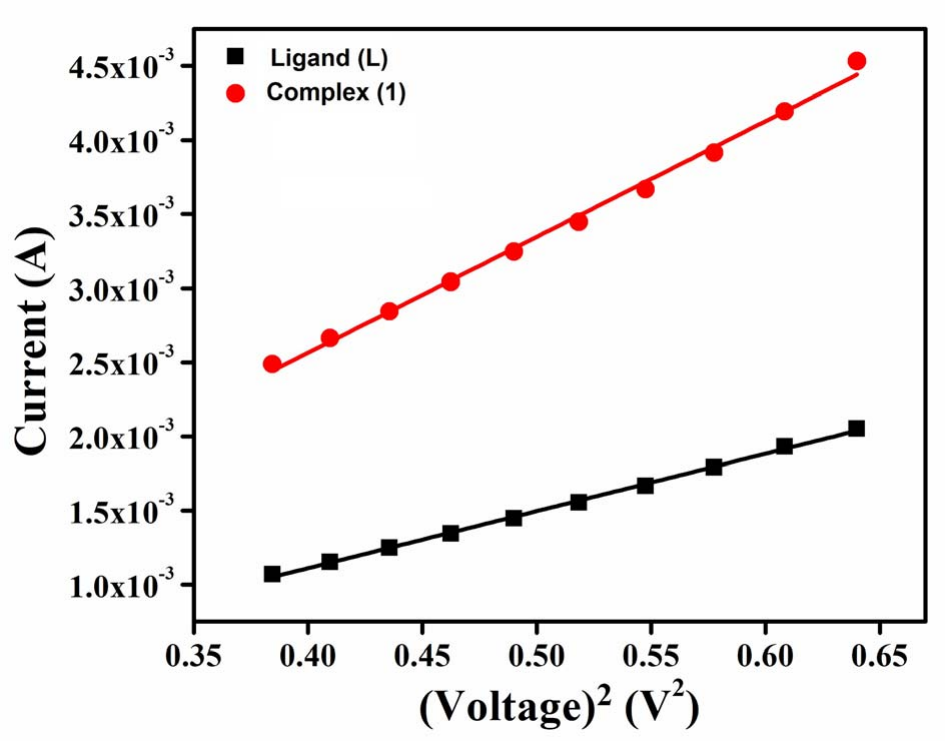
*I vs. V* ^2^ plot of the SCLC region under dark conditions for H_4_L and complex 1.

The space charge limited current (SCLC) phenomenon occurs when injected charge carriers surpass intrinsic levels, forming a space charge that limits current flow *via* its own electric field.^85^ Since carrier mobility and transit time are the critical factors in device performance, the Mott–Gurney SCLC equation was applied to the quadratic region of the *I*–*V* curves (Fig. 8) to calculate the carrier mobility.^86,87^8
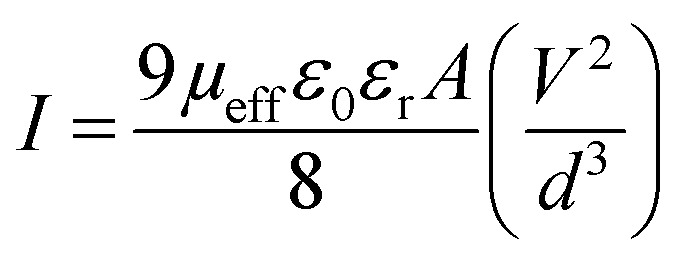


Fascinatingly, the diode constructed with complex 1 showed lower series resistance and barrier height compared to the one made with ligand H_4_L. This improvement can likely be attributed to the incorporation of the metal, with the formation of a 1D polymer through π⋯π interactions. The supramolecular architecture formed through several C–H⋯π and C–H⋯O weak interactions enhances the overall conductivity, allowing for more efficient electron transfer compared to the ligand.

As observed earlier, the superior conductivity of complex 1 is further supported by its optimal band gap, as confirmed by UV-vis analyses and DFT calculations. Thus, the Al complex reduces both the series resistance and the barrier height compared to its ligand H_4_L counterpart. Moreover, the surface conditions and interface quality between the metals (Al^3+^) and the semiconductors greatly affected the device properties. The improved performance of devices with complex 1 emphasizes its possible applicability in organic electronics, showing that both the metal–semiconductor interface quality and surface conditions were important in determining the device characteristics.

**Fig. 9:**
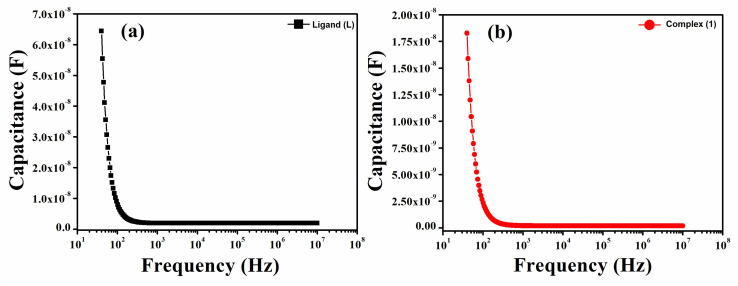
Capacitance *versus* frequency plots for (a) ligand H_4_L and (b) complex 1.

In eqn (8), *I* represents the current, *ε*_0_ is the permittivity of free space, and *µ*_eff_ denotes the effective electron mobility, *d* the thickness of the active layer and *ε*_r_ the dielectric constant of the material. The dielectric constant is determined from the capacitance–frequency (*C*–*f*) plot shown (Fig. 9), utilizing the following formula.^88^9
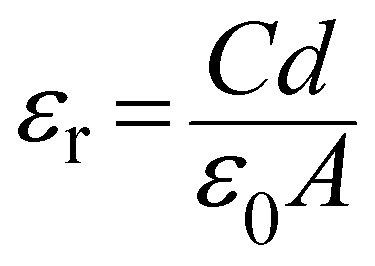


The time required for a charge carrier to cross the distance between the anode and cathode is known as the transit time (*τ*). This value accounts for the cumulative time an electron spends as a free carrier plus its residence time in traps.^89^ This duration (*τ*) can be calculated using the following relationship.^90^10
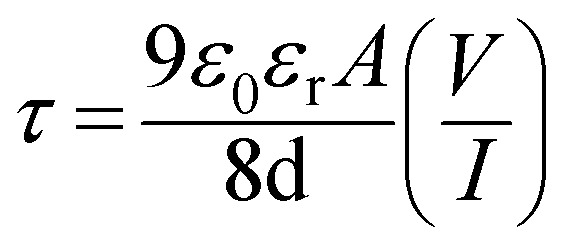


The carrier mobility of complex 1 (3.83 × 10^−5^ m^2^ V^−1^ s^−1^) is significantly higher than that of ligand H_4_L (1.79 × 10^−6^ m^2^ V^−1^ s^−1^) under dark conditions. Although extended transit times typically raise trapping probabilities, the enhanced carrier mobility in these materials reverse that trend.^88^ These parameters, including mobility and transit time, are listed in Table 3.

**Table 3:** Charge transport parameters measured under dark conditions

Sample	Measured condition	Effective carrier mobility (*µ*_eff_) m^2^ v^−1^ s^−1^	Transit time (*τ*) (s)
Ligand H_4_L	Dark	1.79 × 10^−6^	3.92 × 10^−7^
Complex 1	Dark	3.83 × 10^−5^	1.83 × 10^−8^

**Fig. 10:**
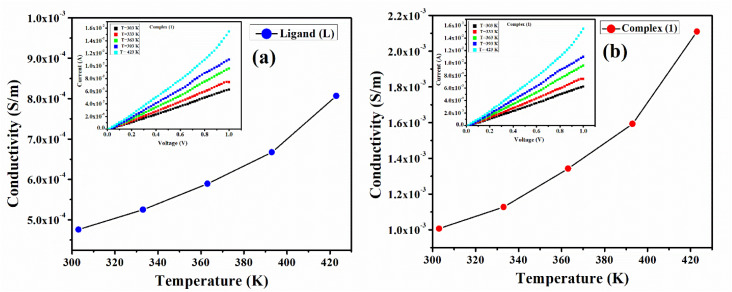
Temperature dependent conductivity plots for (a) ligand H_4_L and (b) complex 1 (inset: plot of current *vs.* voltage at different temperature).

The temperature dependent electrical conductivity measurements of ligand H_4_L and complex 1 were carried out over a temperature range of 303–423 K to verify their semiconducting behavior.^91^ The results clearly show an increase in conductivity with increasing temperature, confirming their semiconducting nature (Fig. 10).

**Fig. 11:**
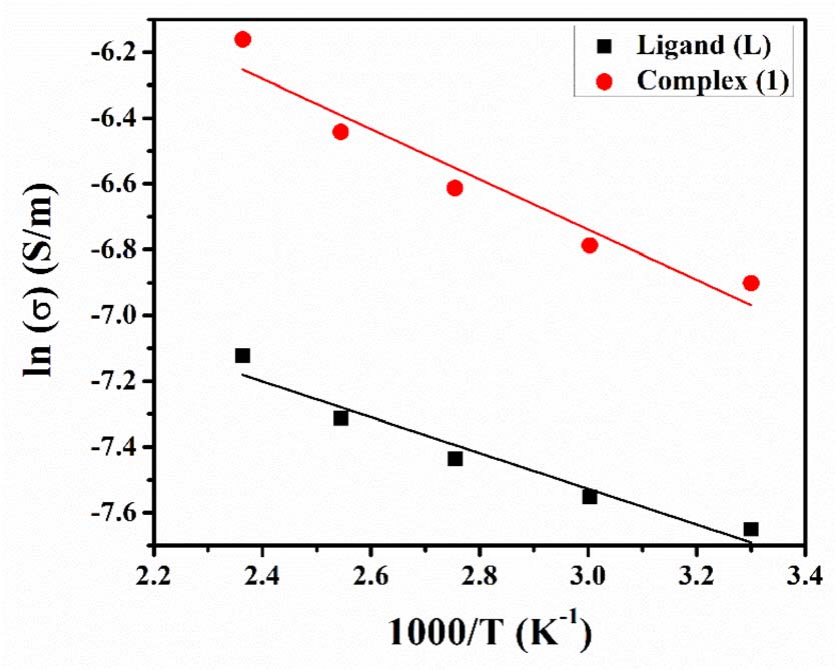
Arrhenius plot for estimation of activation energies for ligand H_4_L and complex 1.

Next, the conductivity data were analyzed using the Arrhenius equation,

11 *σ* = *σ*_0_ exp[−*E*_a_/*k*_B_*T*]

where *E*_a_ is the thermal activation energy, *T* is the temperature, *k*_B_ is the Boltzmann constant and *σ*_0_ is a constant. The linear relationship of the ln(*σ*) *vs.* 1000/*T* plot indicates thermally activated charge transport (Fig. 11).

From the slope of the fitted line, the thermal activation energies were calculated to be 0.046 eV for ligand H_4_L and 0.065 eV for complex 1. The obtained activation energy values fall within the typical range for semiconducting materials, thereby validating the temperature dependent semiconducting characteristics of the material.

Table 4 presents a comparison of the electrical parameters of complex 1 with those of other metal complexes with different Schiff bases, including transition and non-transition metals, reported in literature. Most of the reported complexes exhibit conductivity in the dark in the range of 8.80 × 10^−7^–3.69 × 10^−4^, while complex 1 exhibits a relatively higher conductivity of 8.70 × 10^−4^ S m^−1^. This table also demonstrates that complex 1 is relatively better in terms of overall electrical parameters than other reported complexes. Thus, this kind of material can give inspiration to future researchers in the field of materials science.

**Table 4:** Comparison of the electrical parameters under dark conditions for complex 1 with other related metal complexes reported in the literature^a^

Copper and metal complexes used in Schottky diode	Condition	Device conductivity (S m^−1^)	Rectification ratio	Ideality factor	Barrier height (eV)	Reference
H_4_L^1^	Dark	4.02 × 10^−4^	5.98	2.50	0.62	This work
[Al_2_(L^1^)(H_2_L^1^)]·3H_2_O·DMF	Dark	8.70 × 10^−4^	7.40	2.23	0.59	
H_2_L^2^	Dark	1.05 × 10^−7^	5.27	2.31	0.95	64
[NaAl_2_(L^2^)_2_]·2H_2_O·3.5DMF	Dark	1.04 × 10^−5^	42.0	1.54	0.85	
(Cu_2_L.^3^ 2Py). DMF	Dark	3.49 × 10^−6^	20.45	1.67	0.736	96
[(CdL^4^)_2_(µ1,5-dca)_2_Cd]_*n*_	Dark	3.69 × 10^−4^	48.16	1.97	0.51	23
[Zn(INH)(succ)]_*n*_ [Zn(INH)(fum)]_*n*_ [Zn(INH)(bdc)]_*n*_	Dark	2.26 × 10^−4^	176	1.43	0.368	53
	Dark	1.12 × 10^−4^	80.62	1.94	0.511	
	Dark	1.25 × 10^−4^	81.73	1.86	0.501	
[Zn_4_(bdc)_4_(ppmh)_2_(H_2_O)]_*n*_	Dark	6.20 × 10^−7^	93	3.41	0.60	93
Cu(L^5^)(pyrazine)	Dark	2.34 × 10^−6^	8.49	2.08	0.44	41
[Cu(L^6^)(NCS)]	Dark	6.49 × 10^−4^	23.11	2.29	0.43	56
[Cu_2_(L^7^)_2_]	Dark	2.41 × 10^−7^	32.59	2.05	0.68	94
[(N_3_)Co(L^8^)Na(N_3_)]_*n*_	Dark	8.80 × 10^−7^	25.57	0.27	0.90	61
[Ni_2_(L^7^)Pb(µ-1,3-SCN)_2_(H_2_O)]_2_	Dark	5.74 × 10^−7^	15.66	1.92	0.52	95

a H_2_L^1^ = bis(2-hydroxybenzylidene)-[1,1′-biphenyl]-2,2′-dicarbohydrazide, H_2_L^2^ = bis(2-hydroxynaphthalen-1-ylmethylene)-[1,1′-biphenyl]-2,2′-dicarbohydrazide, H_2_L^3^ = bis(2-hydroxybenzylidene)-1,3-benzenedicarbohydrazone, H_2_L^4^ = *N*,*N*-bis(salicylidene)propane-1,3-diamine, ppmh = *N*-pyridin-2-yl-N0-pyridin-4-ylmethylene-hydrazine, INH = isoniazid, H_2_succ = succinic acid, (H_2_fum) = fumaric acid, H_2_bdc = terephthalic acid, H_2_L^5^ = phthalic acid, HL^6^ = (1-(2-(diethylamino)ethylimino)ethyl)naphthalene-2-ol, H_2_L^7^ = *N*,*N*′-bis(salicylidene)-1,2-ehanediamine, H_2_L^8^ = [*N*,*N*-bis(5-bromosalicylidene)-2,2-dimethyl-1,3-propanediamine].

## 5. UV-vis spectroscopy and band gap

**Fig. 12:**
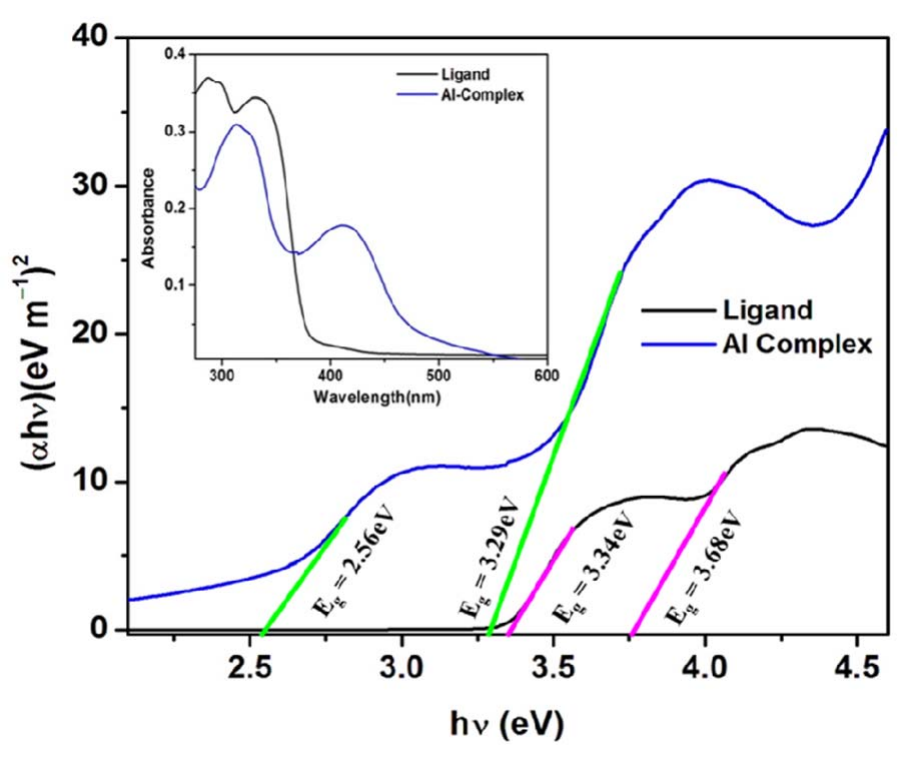
UV-vis absorption spectra (inset) and Tauc plots for ligand H_4_L and complex 1.

To find the optical band gaps of ligand H_4_L and complex 1 in solid states, the UV-vis spectra were recorded and are shown in Fig. 12 (inset). The optical band gaps (*E*_g_) were evaluated using Tauc's equation^92^

**Fig. 13:**
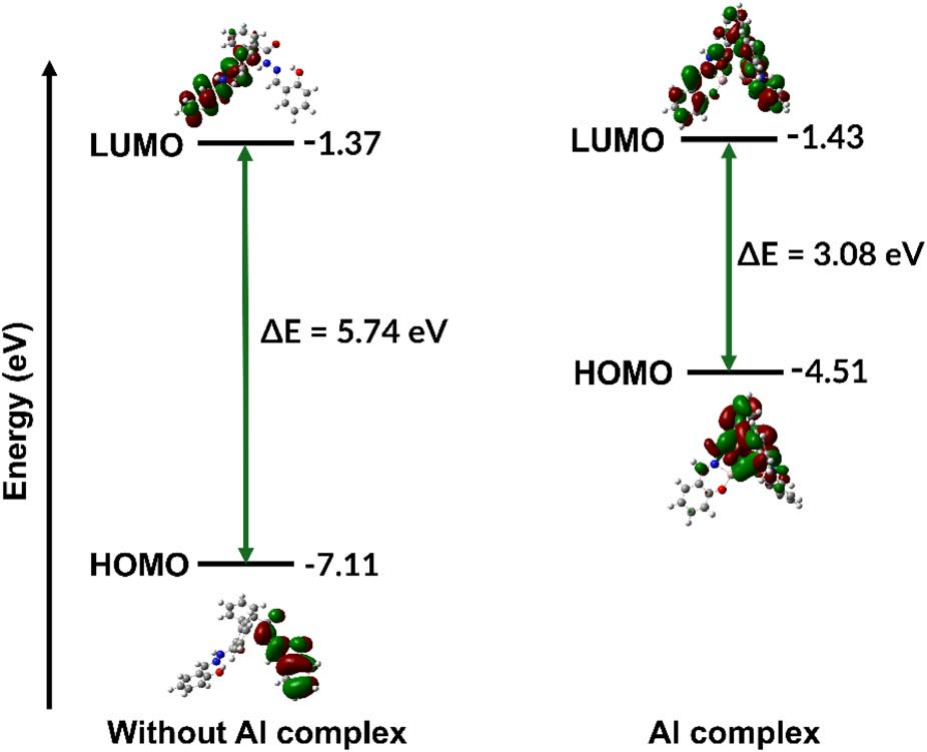
HOMO, LUMO, and HOMO–LUMO energy gap for ligand H_4_L and complex 1 using the B3LYP/6-311G(d) level of theory.

12 (*αhν*)^*n*^ = *A*(*hν* − *E*_g_)

where *α*, *E*_g_, *h*, and *ν* are the absorption coefficient, optical band gap energy, Planck's constant and frequency, respectively. Here, *A* is an arbitary constant which equals 1 for the ideal case, and *n* is the order of energy transition. The values of *n* were taken as 2 and 1/2, corresponding to the allowed direct and indirect electronic transitions, respectively. By extrapolating the linear region of the plot (*αhν*)^2^*vs. hν* (Fig. 13) to *α* = 0 absorption, the values of the direct optical band gap (*E*_g_) of complex 1 were evaluated to be 2.56 and 3.29 eV, suggesting its semi-conducting property, and those of H_4_L as 3.34 and 3.68 eV (Fig. 12 and Table 5).

**Table 5:** Values of optical energy gaps of ligand H_4_L and its Al(iii) complex 1

Compound	Energy gap (eV)
Ligand H_4_L	3.34 and 3.68
Complex 1	2.56 and 3.29

## 6. DFT calculations and HOMO–LUMO energy gap analysis

The DFT calculations for ligand H_4_L and complex 1 were carried out with at the DFT-B3LYP/6-311G(d) level with Gaussian-16 software.^68^Fig. 13 shows the HOMO and LUMO energy values of ligand H_4_L and complex 1. The lower HOMO–LUMO gap of complex 1 (3.08 eV) compared to that of the ligand (5.74 eV) is in agreement with the greater conductivity of the former. It is noteworthy that the HOMO–LUMO energy gaps obtained from the DFT calculations slightly deviate from the experimental values (Table 5). Such differences could be attributed to the fact that the experimental values for the samples were obtained in the solid phase, while the theoretical values were predicted for an isolated metal complex, ignoring the interactions observed in the solid state.

**Fig. 14:**
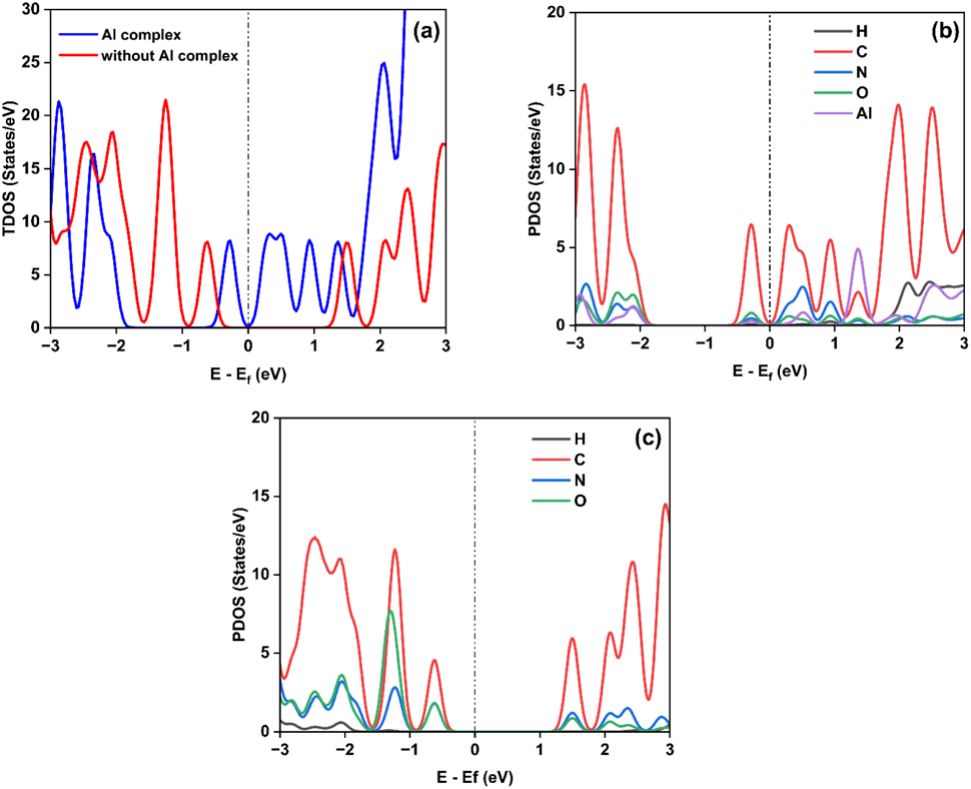
(a) Total density of states (TDOS) spectra of ligand H_4_L and complex 1, and the partial density of states (PDOS) spectra for (b) complex 1 and (c) ligand H_4_L. The black dotted line indicates the Fermi level (*E*_F_).

The semiconducting nature of complex 1 is also confirmed from the density of states (DOS) calculations, as shown in Fig. 14.

In the DOS spectra, there is a significant energy gap between the valence (VB) and conduction band (CB), indicating the semiconductor nature of complex 1 and ligand H_4_L. Additionally, it was observed that complex 1 exhibits a higher conductivity (band gap less than 1 eV) than ligand H_4_L (greater than 1 eV). Additionally, the computed PDOS shows the contributions of individual atoms to the electronic behavior of the ligand and its Al(iii) complex. Importantly, the individual atom contribution for complex 1 is found to be higher than that of ligand H_4_L.

## 7. Conclusions

In conclusion, we have reported the structural characterization of the novel six-coordinated dinuclear double-stranded helicate [Al_2_(L)(H_2_L)]·3H_2_O·DMF (1) containing a biphenyl derived amido Schiff base ligand, bis(2-hydroxybenzylidene)-[1,1′-biphenyl]-2,2′-dicarbohydrazide (H_4_L). The crystal structure of complex 1 shows several types of intra/inter-molecular H-bonds and weak interactions, including C–H⋯O, C–H⋯π and π⋯π stacking, which stabilize the crystal lattice to give rise to a supramolecular polymeric structure. Furthermore, the measured electrical conductivity of complex 1 is double that of the ligand H_4_L, which could be explained by the extended polymeric architecture of H-bonds and several types of weak interactions. The optical band gaps of complex 1 in the solid state were determined from the Tauc plot and compared with the theoretical value obtained from DFT calculations, which indicate that complex 1 could be used as a Schottky barrier diode (SBD).

## Conflicts of interest

The authors declare no competing financial interests.

## Supplementary Material

RA-OLF-D6RA03765H-s001

RA-OLF-D6RA03765H-s002

## Data Availability

CCDC 1990271 for ligand H_4_L and 2515568 for the Al(iii) complex (complex 1) contain the supplementary crystallographic data for this paper.^97*a*,*b*^ Supplementary information (SI) is available. See DOI: https://doi.org/10.1039/d6ra03765h.
