# Supplementary material for: An electrically conductive dinuclear double helicate Al(iii) complex for the fabrication of a Schottky diode
Source: RSC Adv. 2026 Jul 22. Online ahead of print. doi: 10.1039/d6ra03765h (PMC13390602; doi:10.1039/d6ra03765h)
Supplement: RA-OLF-D6RA03765H-s001 [file RA-OLF-D6RA03765H-s001.pdf]

Supplementary Information (SI) for RSC Advances.
This journal is © The Royal Society of Chemistry 2026

An Electrically Conductive Dinuclear Double Helicate Al(III) Complex for the Fabrication of Schottky Diode

Md Hasan Raja,[a] Sayantan Sil,[b] Samim Ahmed,[a] Ramjan Sk,[c] Md Rafikul Alam Mondal,[a] K. Mohamed Yusuf Baig,[d] Manash Pratim Sarmah,[e] Nargis Khatun,[f] Partha Pratim Ray,[c]* Goutam Kumar Kole,[d]* Manabendra Sarma,[e]* A. K. M. Maidul Islam,[f]* Ennio Zangrando,[g]* Md. Akhtarul Alam[a]*

[a]Department of Chemistry, Aliah University, Action Area IIA/27, New Town, Kolkata-700160

[b]Department of Basic Science and Humanities, Institute of Engineering and Management, University of Engineering and Management, Kolkata, University Area, Action Area III, B/5, New Town, Kolkata 700160, India.

[c]Department of Physics, Jadavpur University, Kolkata 700032, India.

[d]Department of Chemistry, SRM Institute of Science and Technology, Kattankulathur, Tamil Nadu 603203, India.

[e]Department of Chemistry, Indian Institute of Technology, Guwahati, Assam - 781039

[f]Department of Physics, Aliah University, Action Area IIA/27, New Town, Kolkata-700160

[g]Department of Chemical and Pharmaceutical Sciences, University of Trieste, Via L. Giorgieri 1, 34127 Trieste, Italy

E-mail: alam_iitg@yahoo.com and alam@aliah.ac.in, partha@phys.jdvu.ac.in, goutamks@srmist.edu.in and gkkole@gmail.com, ezangrando@units.it, maidul79@gmail.com and msarma@iitg.ac.in

Table of Contents

1. **Ligand H_4L……………………………………………………..S3**
2. **Crystal structure……...………………S4**
3. **FT-IR spectra …………..........................……………………S8**
4. **Mass spectra……..………………..….S9**
5. **Powder X-ray diffraction (PXRD)……...………….............……S10**
6. **TGA plot ………………………………………………………S11**
7. **Table for crystal structure data……………………........................S12**

1. Ligand H_4L:

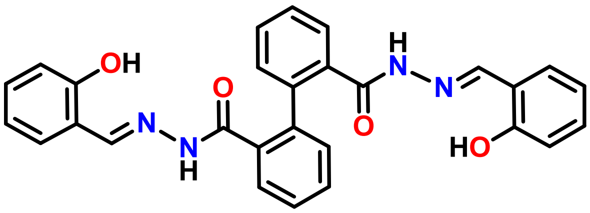

Figure S1. Molecular structure of ligand H_4**L**

2. Crystal structure:

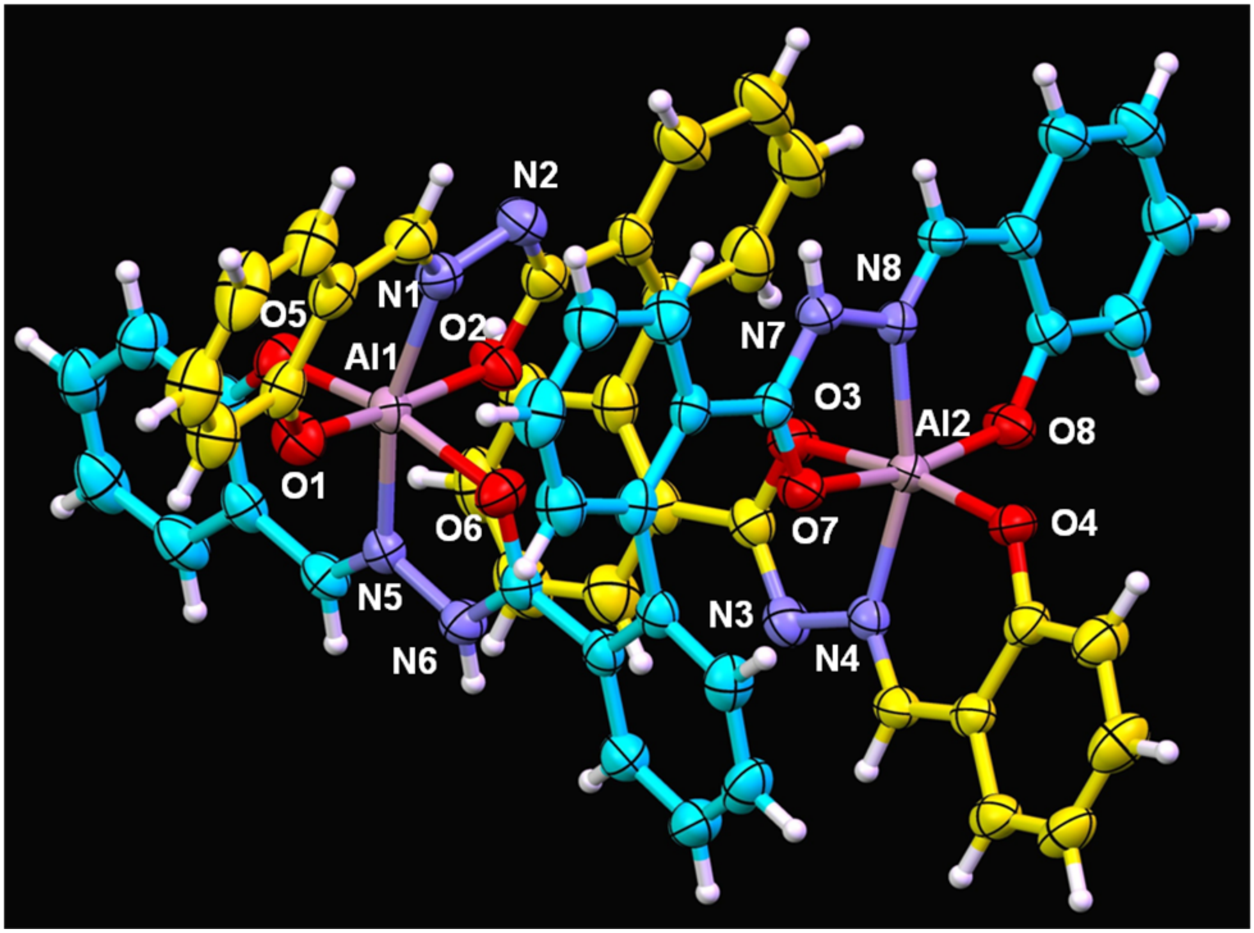

Figure S2. ORTEP representation of complex **1** (probability ellipsoids at 30%) with atom-numbering scheme of coordinating atoms. The two ligands are indicated in different colour. (lattice DMF and water molecules not shown for clarity).

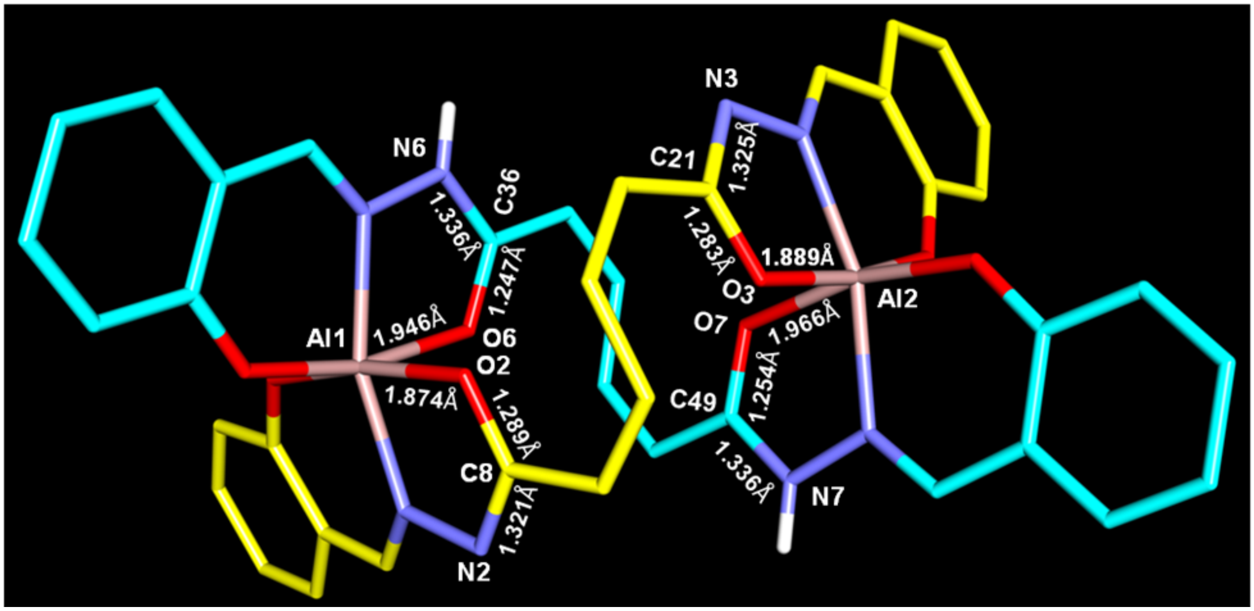

Figure S3. Bond distances of amide oxygen and nitrogen atoms around the Al atoms in complex **1**.

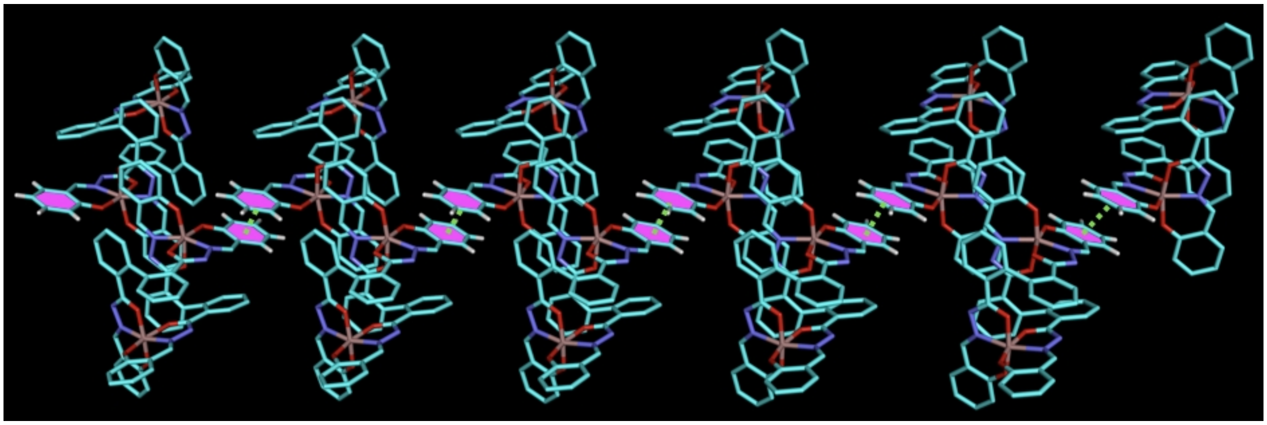

Figure S4. π...π interaction between two dimeric units of complex **1**.

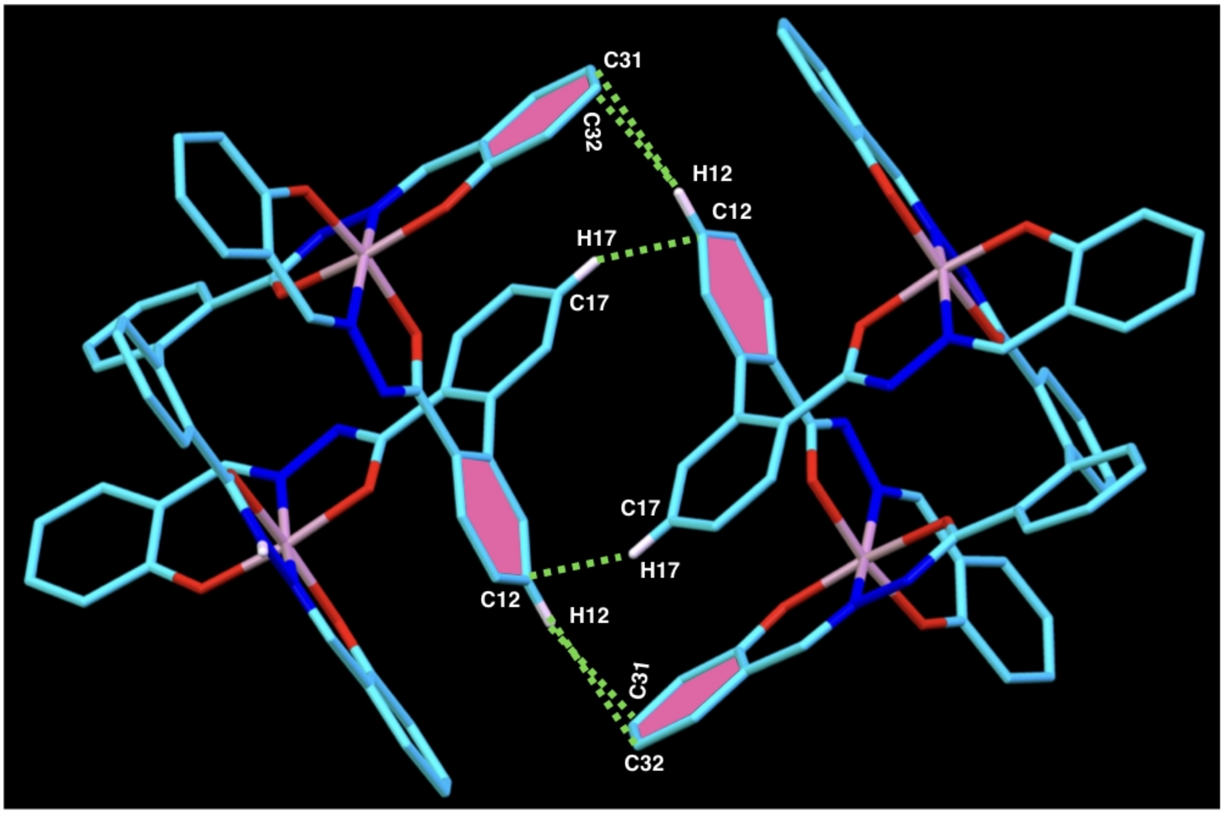

Figure S5. C-H...π interaction between two dimeric units of complex **1**.

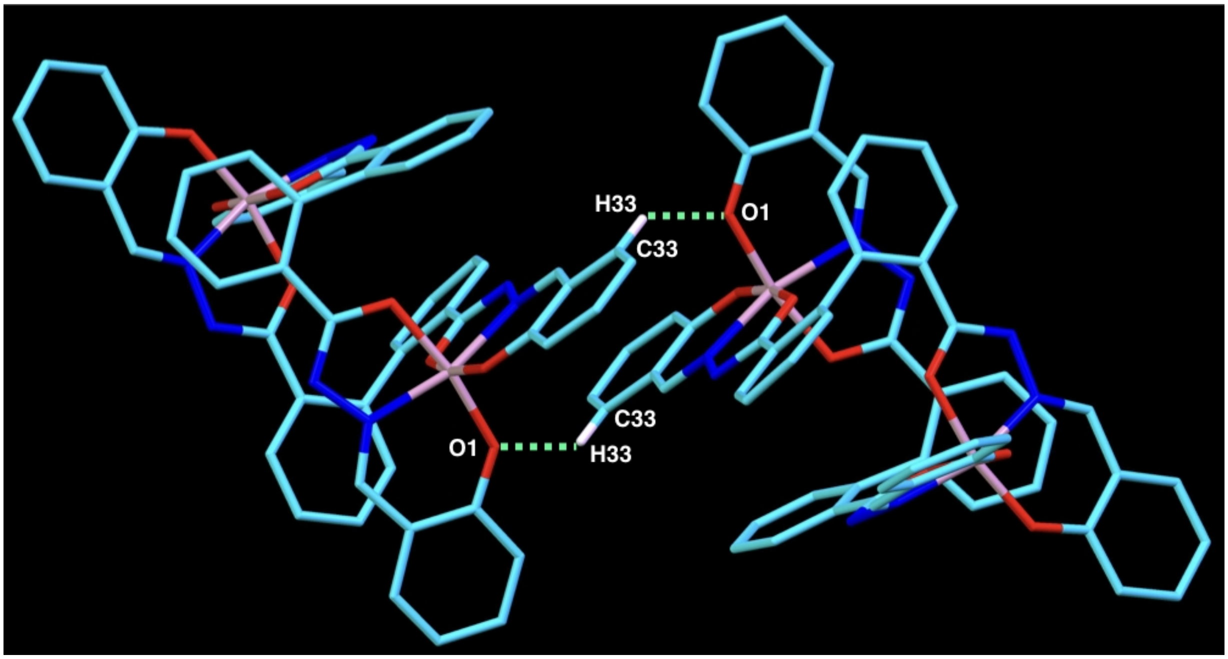

Figure S6. C-H...O interaction between two dimeric units of complex **1**.

3. FT-IR spectra:

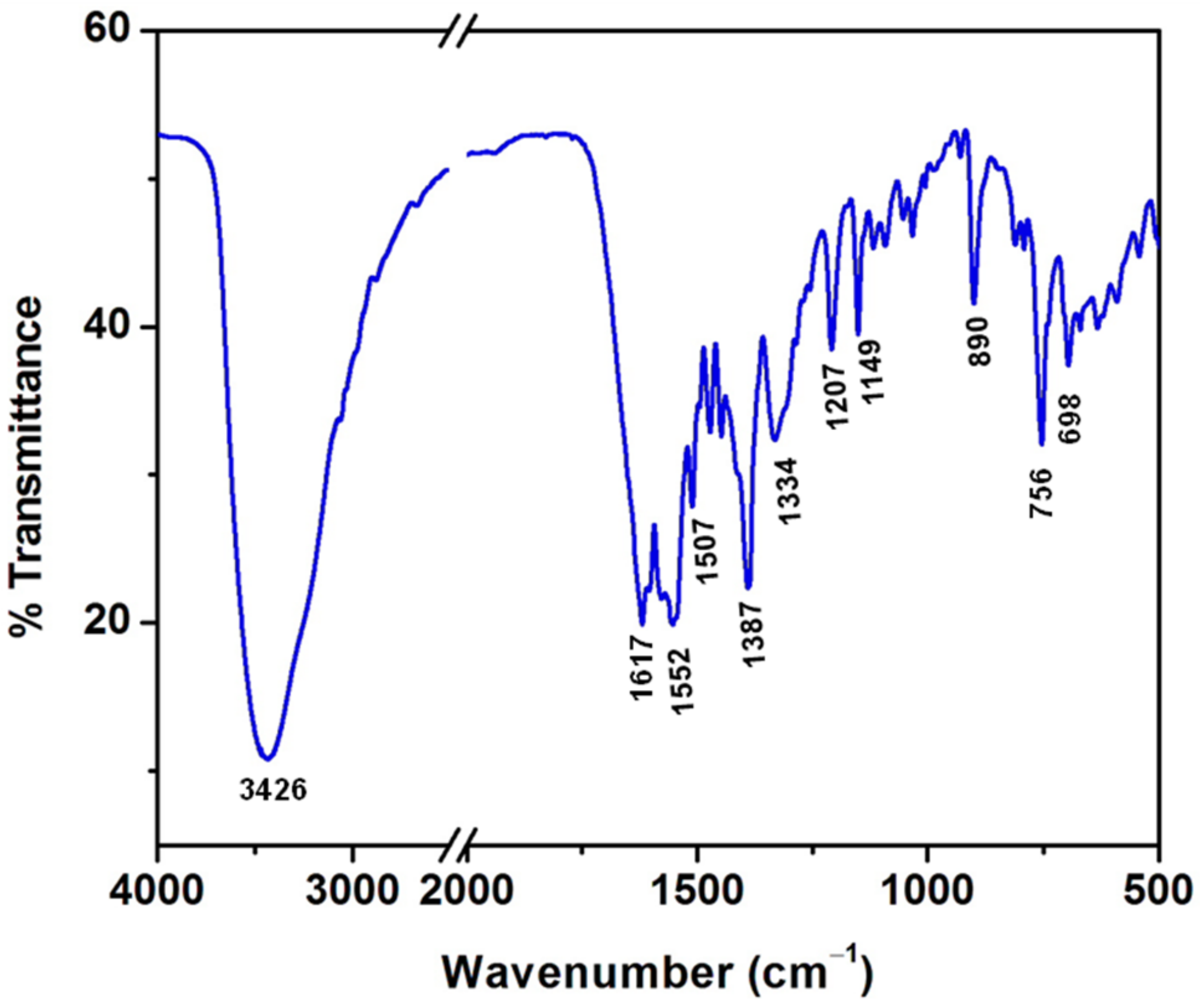

Figure S7. FT-IR spectra complex **1**.

4. Mass spectra:

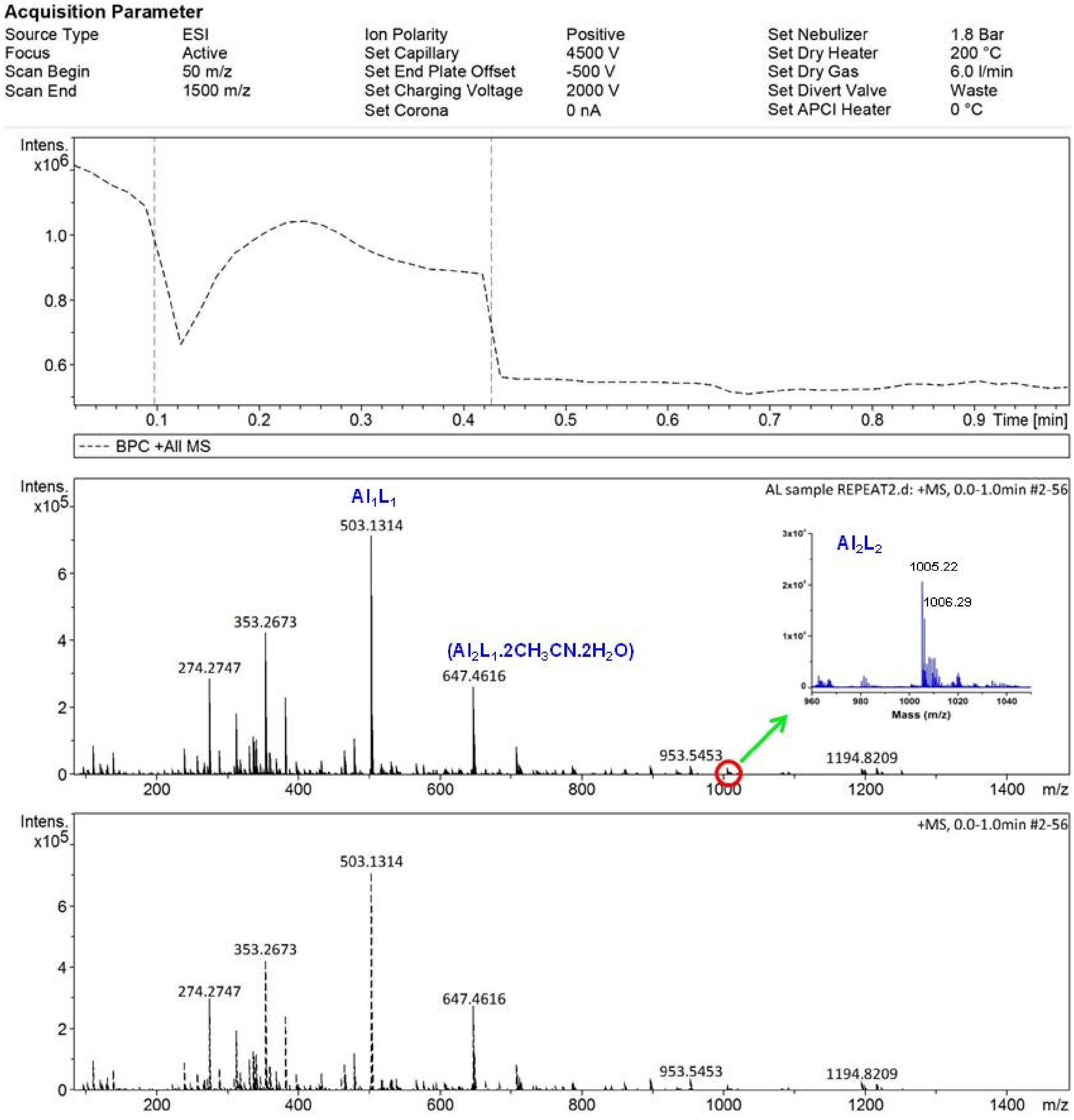

Figure S8. Mass spectrum of complex **1**.

5. Powder X-ray diffraction (PXRD):

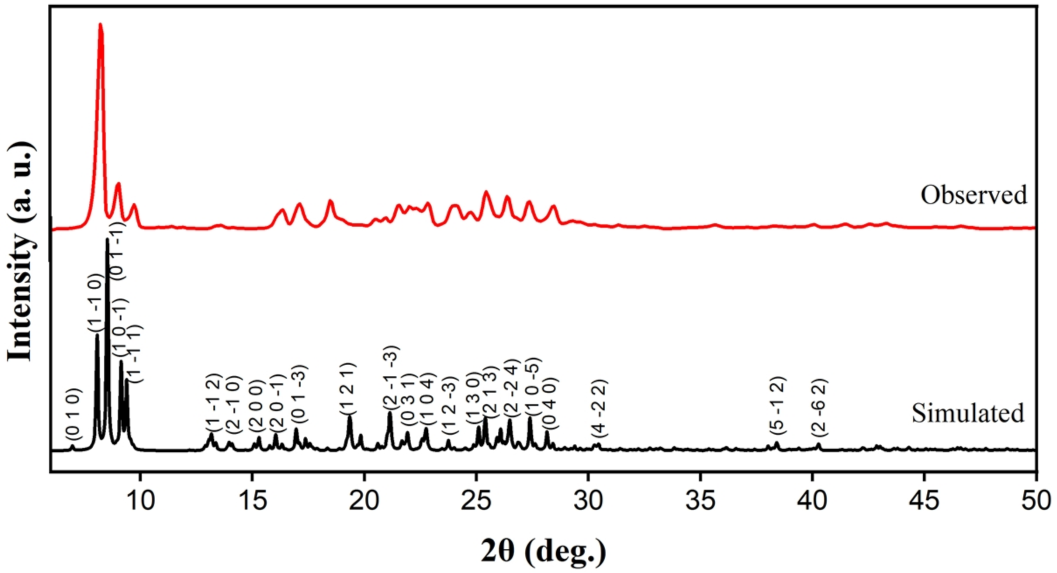

Figure S9. Comparison of PXRD patterns of complex **1** (red) with the simulated pattern from the single crystal structure of complex **1** (black).

6. TGA plot:

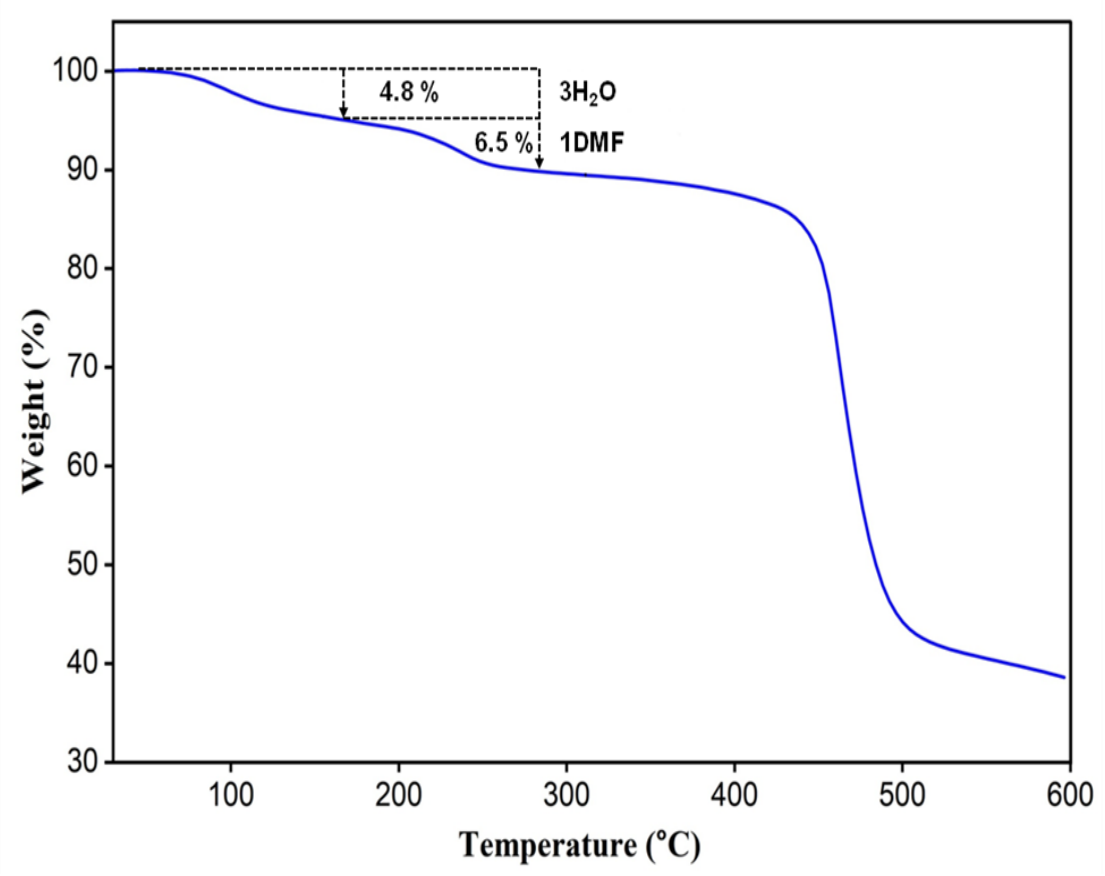

Figure S10. TGA plot of complex **1**

7. Table for crystal structure data:

Table S1. Crystal data and structure refinement for Complex **1**

**Identification Code**	**Complex 1**
CCDC	2515568
Empirical formula	$C_{59}H_{51}Al_2N_9O_{12}$
Formula weight	1132.04
Temperature/K	296 K
Crystal system	triclinic
Crystal colour	yellow
Space group	P -1
α/°	92.294(8)
β/°	90.594(9)
γ/°	113.018(7)
Volume/Å^3	2682.9(11)
Z	2
ρ_{calc}g/cm^3	1.401
μ/mm^{-1}	0.129
F(000)	1180
Crystal size/mm^3	12.583×13.781× 16.830
Radiation	MoK\a
Index Ranges	
h	15
k	17
l	20
Reflections collected	10561
Independent reflections	0.0407, 0.0645
Data/restraints/parameters	10561/ 9/ 761
Goodness-of-fit on F^2	1.104
Final R indexes [I>=2σ (I)]	0.0598
Final R indexes [all data]	0.0735

Table S2. Selected bond distances (Å) and angles (°) of complex **1**

Bond distances of Complex **1**			
Al(1)-O(1)	1.8221(19)	Al(2)-O(3)	1.8890(19)
Al(1)-O(2)	1.8742(19)	Al(2)-O(4)	1.8312(18)
Al(1)-O(5)	1.8162(19)	Al(2)-O(7)	1.9664(18)
Al(1)-O(6)	1.9466(18)	Al(2)-O(8)	1.8222(18)
Al(1)-N(1)	1.982(2)	Al(2)-N(4)	1.967(2)
Al(1)-N(5)	2.013(2)	Al(2)-N(8)	1.999(2)
**Bond angles of Complex 1**			
O(5)-Al(1)-O(6)	167.19(9)	O(4)-Al(2)-O(8)	90.85(9)
O(5)-Al(1)-O(1)	92.04(9)	O(4)-Al(2)-O(7)	90.10(8)
O(6)-Al(1)-O(1)	89.15(9)	O(8)-Al(2)-O(7)	167.32(8)
O(5)-Al(1)-O(2)	93.58(9)	O(4)-Al(2)-N(8)	100.00(8)
O(6)-Al(1)-O(2)	87.28(9)	O(8)-Al(2)-N(8)	89.48(8)
O(1)-Al(1)-O(2)	169.69(9)	O(7)-Al(2)-N(8)	77.91(8)
O(5)-Al(1)-N(1)	101.52(9)	O(4)-Al(2)-O(3)	170.56(8)
O(6)-Al(1)-N(1)	91.20(8)	O(8)-Al(2)-O(3)	93.11(9)
O(1)-Al(1)-N(1)	91.19(9)	O(7)-Al(2)-O(3)	87.92(8)
O(2)-Al(1)-N(1)	79.22(8)	N(8)-Al(2)-O(3)	88.61(8)
O(5)-Al(1)-N(5)	89.77(8)	O(4)-Al(2)-N(4)	91.24(8)
O(6)-Al(1)-N(5)	77.49(8)	O(8)-Al(2)-N(4)	105.75(8)
O(1)-Al(1)-N(5)	100.76(9)	O(7)-Al(2)-N(4)	86.87(8)
O(2)-Al(1)-N(5)	87.91(8)	N(8)-Al(2)-N(4)	161.00(9)
N(1)-Al(1)-N(5)	163.30(9)	O(3)-Al(2)-N(4)	79.43(8)

Table S3. Al-O, amide C=O and amide C-N bond lengths (Å) in complex **1**

Aluminium atom	-C=O···Al^{3+}	-C=O	Carbonyl-amide -C-NH
Al1	1.8742(19)	1.289(3)	1.325(3)
	1.9466(18)	1.247(3)	1.338(3)
Al2	1.8890(19)	1.283(3)	1.325(3)
	1.9664(18)	1.254(3)	1.336(3)

Table S4. Amide C=O and amide C-N bond lengths (Å) in ligand **L**

Atomic number	-C=O	Atomic number	Carbonyl-amide -C-NH
C8=O2	1.222(2)	C8-N3H	1.339(3)
C21=O3	1.233(2)	C21-N1H	1.334(2)

Table S5. Hydrogen bond parameters (Å/°) of complex **1**.

D-H···A	D-H	H···A	D···A	D-H···A
O12-H12A···N3	0.85	2.005	2.818	159
O12-H12B···O9	0.85	2.217	2.950	144
O10-H10B···O12	0.85(11)	1.814	2.651	167
O10-H10A···N2	0.85(11)	2.096	2.942	173
O11-H11A···O10	0.94(4)	1.815	2.732	162
O11-H11B···O4	0.79(4)	2.217	2.982	165
N6-NH6···O9	0.87(3)	2.018	2.875	168
N7-NH7···O11	0.85(3)	1.918	2.760	169

Table S6. C−H···O weak interactions in complex **1**

D-H…A	D-H	H…A	D…A	D-H…A
C33-H33…O1	0.93	2.680	3.480	138
C38-H38…O9	0.93	2.59	3.503	167
C57-H57…O5	0.93	2.610	3.481	156
C39-H39…O10	0.93	2.641	3.446	145
C10-H10…O10	0.93	2.669	3.573	164

Table S7. C−H···π weak interactions

Complex **1**			
C−H···π(C)	C···π(C) distance (Å)	H···π(C) distance (Å)	C−H···π(C) angle (°)
C12−H12···C31	3.492	2.571	171.06
C12−H12···C32	3.709	2.880	149.22
C17−H17···C11	3.662	2.858	145.46

Table S8. π ···π weak interactions

Complex **1**

π···π interaction	distance (Å)
Cg(C23−C28)···Cg(C23−C28)	3.927

centroid of C23-C28 of one phenyl ring and C23- C28 of another unit containing phenyl ring are formed π-π- interaction distance= 3.927
